# @myTabu—A Placebo Controlled Randomized Trial of a Guided Web-Based Intervention for Individuals Who Sexually Abused Children and Individuals Who Consumed Child Sexual Exploitation Material: A Clinical Study Protocol

**DOI:** 10.3389/fpsyt.2020.575464

**Published:** 2021-01-08

**Authors:** Peter Fromberger, Sonja Schröder, Louisa Bauer, Bruno Siegel, Safiye Tozdan, Peer Briken, Claudia Buntrock, Sonja Etzler, Martin Rettenberger, Andreas Leha, Jürgen L. Müller

**Affiliations:** ^1^Clinic for Psychiatry and Psychotherapy-Forensic Psychiatry, University Medical Center Göttingen, Georg-August-University Göttingen, Göttingen, Germany; ^2^Center for Psychosocial Medicine, Institute for Sex Research, Sexual Medicine & Forensic Psychiatry, University Medical Center Hamburg-Eppendorf, Hamburg, Germany; ^3^Department of Clinical Psychology and Psychotherapy, Friedrich-Alexander University Erlangen-Nuremberg, Erlangen, Germany; ^4^Centre for Criminology, Wiesbaden, Germany; ^5^Department of Medical Statistics, University Medical Center Göttingen, Georg-August-University Göttingen, Göttingen, Germany

**Keywords:** eHealth, child sexual abuse (CSA), child exploitation material offenders, web-based intervention, randomized placebo controlled trial

## Abstract

**Background:** There is a high demand for evidence-based and cost-effective treatment concepts for convicted individuals who sexually abused children (ISAC) and individuals who consumed child sexual exploitation material (ICCSEM) under community supervision (CS). The @myTabu-consortium developed a guided web-based intervention for convicted ISAC and ICCSEM under CS consisting of six online modules targeting psychological meaningful risk factors. The study aims to evaluate the effectiveness of this guided web-based intervention in reducing dynamic risk factors and the risk to re-offend compared to a placebo condition. Furthermore, these dynamic risk factors are measured before and after every module to evaluate their individual effectiveness to reduce the respective risk factor as well as risk to re-offend. This clinical trial protocol describes the planned methods as well as the intervention concept.

**Methods:** The methodological design is a placebo controlled randomized add-on trial (*N* = 582) with follow-ups at 8 points in time. The placebo condition controls for attention and expectation effects and comprises the same amount of modules with a comparable temporal effort as the experimental intervention. The trial is conducted as an add-on to community supervision as usually done. Primary outcomes are dynamic risk factors assessed by self-report risk assessment tools and officially recorded re-offenses.

**Discussion:** To the best of our knowledge, the study is the first to compare the (cost-) effectiveness of a guided web-based intervention for convicted ISAC and ICCSEM under community supervision against a placebo condition. Methodological limitations (e.g., potential ceiling- or volunteers-effects) are discussed.

**Clinical Trial Registration**: German Clinical Trial Register (DRKS 00021256). Prospectively registered: 24.04.2020.

## 1. Introduction

Many convicted individuals who sexually abused children (ISAC) and individuals who consumed child sexual exploitation material (ICCSEM) remain untreated in Germany. This originates both from only having few possibilities for community-based treatment and an increase of convicted ISAC and ICCSEM under community supervision (CS) within the last years [a 48% increase from 2007 to 2013 (1)]. Outpatient treatment centers for people fulfilling the criteria of a pedophilic disorder, who have not yet abused a child, do not accept already convicted ISAC and ICCSEM under CS into their treatment programs in most cases (2). Neither do most psychotherapists accept convicted ISAC and ICCSEM under CS for treatment because of the potential risk to re-offend or other reasons (e.g., in order to avoid contact between offenders and victims). For example, 87% of psychotherapists would not provide treatment for ISAC, whereas only 2.7% would do so (3). Thus, the majority of convicted ISAC and ICCSEM under CS have difficulties to receive professional treatment during CS, especially in rural areas. Beside the immense burden to the individuals, child abuse results also in trauma follow-up costs of economically relevant magnitude for the German society (4). Financial consequences of sexual offenses include e.g., victim-related costs (e.g., medical and psychological services to aid victim recovery), offender-related costs (e.g., costs of law enforcement and incarceration), and intangible costs (e.g., the fear that forces victims to schedule normal activities around issues of safety) (5). In summary, there is a high demand for evidence-based and cost-effective treatment concepts for convicted ISAC and ICCSEM under CS. This clinical trial protocol describes the planned methods as well as the intervention concept of a placebo controlled randomized trial to test a guided web-based intervention for convicted ISAC and ICCSEM.

### 1.1. Effectiveness of Treatment Programs

Studies evaluating the outcome of treatment programs for individuals who have sexually offended often suffer from methodological problems. Especially the lack of an adequate control group as well as the fact that most studies are under-powered are criticized (6). Despite these criticisms, in face-to-face (f2f) treatment for ISAC, meta-analyses demonstrated that cognitive-behavioral treatment programs based on the Risk-Need-Responsivity [RNR; (7)] model are most effective in reducing re-offenses of individuals who have sexually offended with a small but stable effect size (8–10). Kim et al. reviewed all meta-analyses until 2009 and report a mean effect size of *d*=0.36. Furthermore, this meta-analyses showed that effectiveness of treatment differs not only with regard to treatment type but also with regard to treatment setting: community treatment provides a higher effectiveness (*d* = 0.33) than institutional treatment programs (*d* = 0.20) (10). Additionally, Schmucker and Lösel showed that individual treatment seems to be more effective than group therapy and that community treatment provides higher effectiveness than institutional treatment (11). Grønnerød et al. reviewed the effectiveness of treatment programs especially for ISAC. They reported an average recidivism rate of 18% for treated and 20% for untreated ISAC (12). In summary, the majority of meta-analyses report a small but clinically significant behavioral change caused by treatment. Officially registered new offenses are the standard concerning the primary outcome criterion for treatment programs for individuals who have sexually offended. However, officially registered recidivism is not a very sensitive measure for treatment effects (11, 13) due to several reasons. First, recidivism rates are generally low [around 6% in (14)] and since many delinquent behaviors might not be detected and registered by the law enforcement authorities, this results in a low test power for recidivism change and a high likelihood of type-2 errors. Second, there is a time lag between the manifestation of deviant behavior and its official registration deferring the adequate time frame of its assessment. Finally, recidivism rates represent a distal outcome and do not provide any information about the specific processes of risk reduction through treatment. Thus, some authors highlight the importance to examine within-treatment changes of risk factors associated with recidivism (15, 16) and the use of standardized risk assessment tools instead of focusing on officially recorded re-offenses (17, 18). Olver and Wong as well as Olver and Stockdale proposed to consider questionnaires correlated with meaningful risk factors and structured dynamic risk assessment tools in order to assess treatment change (16, 19). Self-report measures offer several advantages in the assessment of psychological constructs regrading dynamic and acute risk factors. They are highly economic, standardized, and it is possible to implement them into online settings. Studies revealed that individuals convicted of (sexual) offenses disclose a relatively high amount of sexual aggression in paper-and-pencil surveys (20) as well as web-based surveys (21). Additionally, self-report instruments are used to assess different kinds of risk factors as well as deviant behaviors with no time delay directly after its manifestation. For the purpose of tracking differential treatment effects, Olver and Wong highlight the necessity of considering the pre-treatment levels of participants with regard to risk factors, since ISAC have different potential to change depending on their risk level and therefore can differently benefit from treatment. Assessing pre-post treatment changes with questionnaires resulted in effect sizes ranging from *d* = 0.25 to 1.34 (17). Studies assessing pre-post treatment changes with standardized risk assessment tools report, e.g., effect sizes of *d* = 0.77 for the Stable-2007 (22) and *d* = 0.35 for the Acute-2007 (23).

### 1.2. Web-Based Interventions for Convicted ISAC and ICCSEM Under CS

Over the last few years, guided web-based interventions showed high efficacy in the treatment of psychiatric disorders (24). Web-based interventions demonstrated similar effects to f2f psychotherapy when directly compared (25). Meta-analytic findings suggest that stand-alone web-based interventions with guidance tend to have greater effect sizes than web-based interventions without professional assistance. Web-based interventions with professional assistance showed smaller attrition rates, a higher number of completed modules, and larger symptom reduction in users suffer from, e.g., depression, anxiety disorders, and eating disorders (26–30). Despite the qualitative and quantitative reduction of overall therapeutic contact in web-based interventions (e.g., absence of social and non-verbal signals), studies seem to indicate that a comparable perceived quality of the therapeutic alliance can be achieved as in f2f settings (31–36). Therefore, it seems reasonable to assume that web-based interventions may be as effective as f2f treatment of ISAC, if they concentrate on (psychological meaningful) risk factors, use evidence-based treatment techniques, follow the RNR principles, are conducted in community programs, and provide professional assistance during the intervention (37). Given the evidence for the effectiveness of cognitive-behavioral treatment programs based on RNR principles, it seems likely that such interventions could also produce significant cost savings. However, empirical evidence on the economic benefits of such a web-based intervention does not exist yet. Moreover, to our knowledge there is only one pilot study, which tested the feasibility of a web-based intervention for ISAC [for an overview see (37, 38)]. Kernsmith and Kernsmith reported on a study utilizing an online support group (comparable to an online forum) for recovering individuals who have sexually offended (39). Even though this intervention cannot be compared directly with @myTabu, it demonstrated the feasibility of using web-based methods with ISAC. For ISAC and ICCSEM an online self-program (“Stop it now!”) exists, developed and provided by the Lucy Faithful Foundation (40). However, to date, the only study published is a pilot study about the Stop it Now! Helpline (41).

### 1.3. Aims and Hypotheses

Until now, to the best of our knowledge, a guided web-based intervention has not been evaluated with convicted ISAC and ICCSEM under CS or evaluated with regard to its (cost-) effectiveness. @myTabu aims to develop and to evaluate the effectiveness and economic benefit of a guided web-based intervention program in reducing dynamic risk factors compared to a placebo condition. Furthermore, it aims to evaluate the effectiveness of the individual online modules within the intervention program with regard to its ability to reduce dynamic risk factors. The @myTabu consortium consists of experts in the treatment of ISAC and ICCSEM (University Medical Center Göttingen, Georg-August-University Göttingen, Clinic for Psychiatry and Psychotherapy-Forensic Psychiatry, Göttingen, Germany; University Medical Center Hamburg-Eppendorf, Center for Psychosocial Medicine, Institute for Sex Research, Sexual Medicine & Forensic Psychiatry, Hamburg, Germany), experts for web-based interventions (Friedrich-Alexander University Erlangen-Nuremberg, Department of Clinical Psychology and Psychotherapy, Erlangen-Nuremberg, Germany), experts in the risk-assessment of ISAC and ICCSEM (Centre for Criminology, Wiesbaden, Germany) as well as legal experts with regard to community supervision (German Police University, Department of Legal and Criminal Sciences, Münster, Germany).

**Figure 1:**
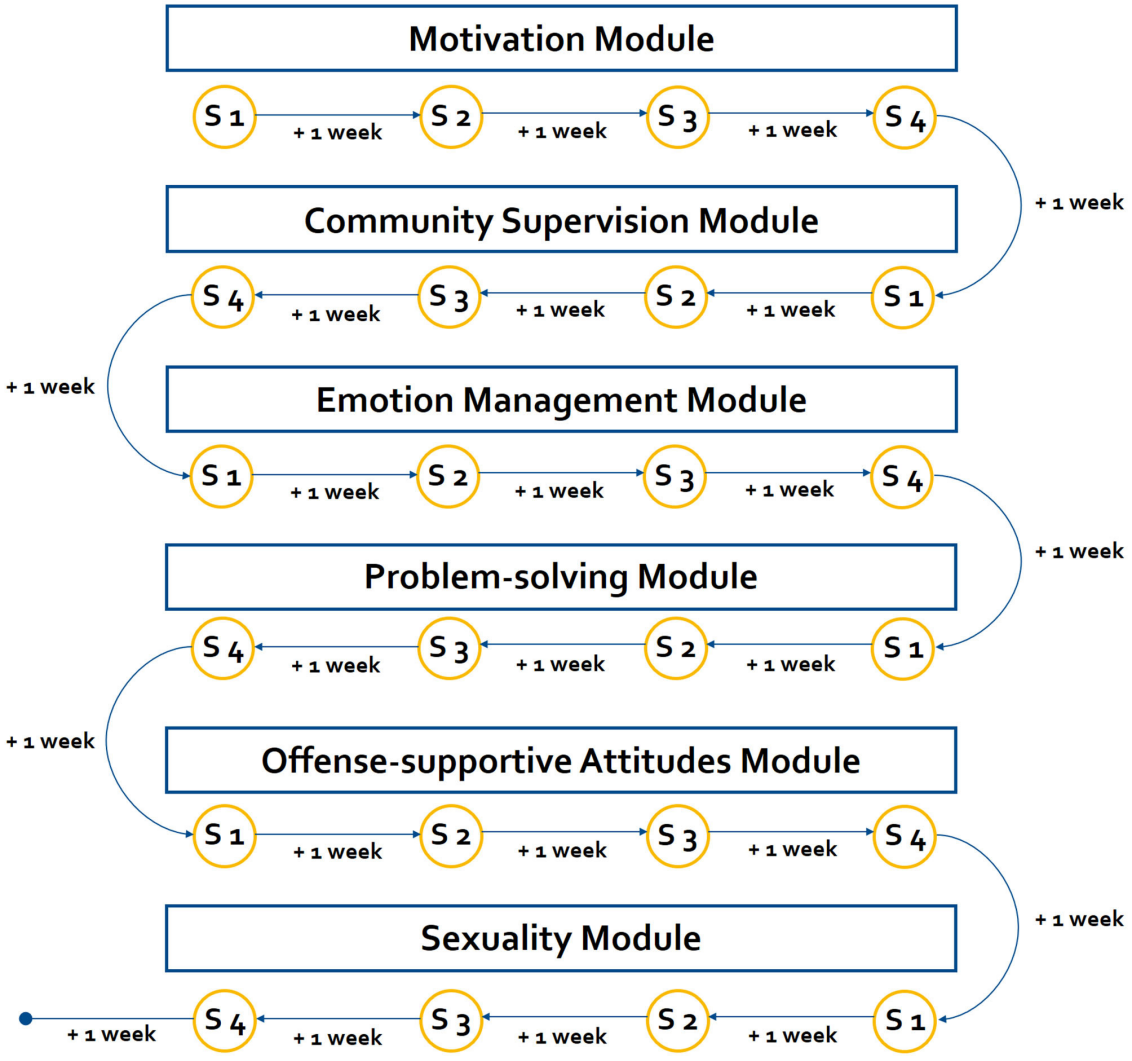
Overview of the web-based intervention. The web-based intervention (control as well as experimental) comprises six modules. Each module consists of four sessions (S). The participants can freely decide when they will work on the session. The next session becomes unlocked, when (1) 1 week has past since last session was completed and (2) all guided-tasks are successfully completed. Thus, participants are able to finish each module within 4 weeks (1 month).

It is expected that convicted ISAC and ICCSEM under CS, who take part in the web-based intervention, show significantly stronger reduction in self-reported dynamic risk factors inter-, and post-treatment controlling for baseline values as well as less officially recorded re-offenses in a 5-years follow-up, compared to an online-based placebo condition. It is further expected that each individual module of the web-based intervention will have positive effects on its targeted risk factors. The web-based intervention comprises six modules (see Figure 1). For the motivation module it is expected that the motivation to take part in the intervention and the motivation to change the own behavior increases. For the supervision module, it is expected that negative social influences and resistance to rules and supervision decrease while interest in emotionally intimate relationships with adults increase. For the emotion management module, it is assumed that impulsivity as well as dysfunctional coping strategies will decrease. The problem solving module is expected to increase the ability to solve problems. For the offense-supportive attitudes module, it is expected that the agreement with cognitive distortions will decrease. Lastly, it is hypothesized that after the sexuality module, the emotional congruence with children, sexual preoccupation and sexual fantasies with children will decrease. Finally, it is hypothesized that there is a positive net economic benefit. It is expected that the gained tangible benefits (e.g., savings in victim- and offender-related costs) and intangible benefits (e.g., health consequences of pain and suffering and possible loss of life) outweigh the costs of delivering the web-based intervention of @myTabu.

## 2. Method and Analysis

### 2.1. Design

Up to now, one of the major criticism in RCTs with convicted ISAC and ICCSEM under CS is that mostly the control group was not treated in any way (6). Thus, the clinical trial is designed as an add-on placebo controlled randomized trial with two parallel conditions: (1) web-based intervention (experimental intervention) and (2) web-based placebo condition (control intervention). The placebo condition controls for attention and expectation effects as well as possible effects of contact with the coach. It comprises the same amount of modules with a comparable time effort as the experimental intervention. The topics covered are unrelated to risk factors. Both conditions will be provided as an add-on to the CS as Usual (CSaU). CSaU comprises all orders of the court and treatment as usual [e.g., f2f contacts with supervision officers (SOs), therapeutic treatment ordered by court, medical treatment].

### 2.2. Participants

Participants include male and female offenders which are under CS (§§56, 57, 68 ff. Strafgesetzbuch, StGB) because of child abuse (§§176, 176a, 176b StGB) or child sexual exploitation material use (§184b StGB). All participants are informed by a scientific staff member and must provide written informed consent before they are included in the clinical trial.

#### 2.2.1. Inclusion Criteria

Inclusion criteria are (1) being under CS due to at least one child abuse (§§176, 176a, 176b StGB) or child sexual exploitation material use (§184b StGB) and (2) being at least 18 years of age.

#### 2.2.2. Exclusion Criteria

Exclusion criteria are (1) a probation period shorter than 6 months at enrollment, (2) no access to a PC, tablet or smart phone, (3) a severe acute psychiatric disorder (e.g., acute psychosis), (4) a severe cerebro-organic disorder, (5) a severe cognitive impairment, (6) withdrawal of the informed consent, and (7) no written informed consent.

#### 2.2.3. Detailed Sample Description

In order to describe the participating sample in more detail, the following questionnaires/structured interviews are applied:

##### 2.2.3.1. Sample Specifications Questionnaire for the Participant (SSQ-P)

The SSQ-P is an online questionnaire asking the participants about their demographics, lifestyle, current and former therapeutic treatments and offending history.

##### 2.2.3.2. Sample Specifications Questionnaire for the SO (SSQ-SO)

The SSQ-SO is an online questionnaire for the SO concerning the current living condition, offending and clinical history, and index offense of the participant.

##### 2.2.3.3. Repeated Sample Specifications Questionnaire for the SO (SSQ-rSO)

The SSQ-rSO is an online questionnaire asking the SO frequently about the participant's conduct during probation period.

##### 2.2.3.4. Sample Specifications Questionnaire based on the Court File (SSQ-CF)

The SSQ-CF is a checklist for coding court files. Information from the court files is coded with the SSQ-CF by the study investigators to determine whether the participant meets the study inclusion criteria and to be able to fill out the Static-99 (42) (only CSOs) or modified Static-99 (43) (only ICCSEM). This includes information on the probation period, index offense, and former offenses. In case that items of the SSQ-CF cannot be filled out adequately by utilizing court files, the participants will be interviewed directly.

##### 2.2.3.5. Patient Health Questionnaire (PHQ-D)

The PHQ-D (44) is a screening tool for mental disorders. Classification of results generally takes place on a syndrome-level. Syndromes examined are: Somatoform disorders, major depressive syndrome, other depressive syndrome, panic syndrome, other anxiety syndromes, eating disorder syndrome, alcohol abuse. Tests are scored using a stencil. Utilizing a sum score for each syndrome, conclusions pertaining to the severity of each syndrome can be drawn. The measure shows good to very good diagnostic validity, especially regarding panic disorder and major depression (45).

##### 2.2.3.6. Revised Scale for Pedophilic Interests (SSPI-2)

The SSPI-2 (46) is a structured rating scale of assessing pedophilic interests based on the offending behavior. It comprises five items (number, age, gender, relationship of victims, and child pornography) and is significantly associated with phallometrically assessed sexual arousal to children. The SSPI-2 is derived from information of the participants as well as the SO.

##### 2.2.3.7. ICD-11-Sexuality-Screener

The ICD-11-Sexuality-Screener (47) is a self-assessment procedure to ascertain indications for the presence of sexual dysfunction, gender dysphoria, or paraphilic disorder. However, the instrument is not suitable for diagnosis. It is assumed that the ICD-11-Sexuality-Screener can identify individuals who have the disorders described above. The ICD-11-Screener is not validated yet.

### 2.3. The Web-Based Intervention @myTabu (Experimental Condition)

**Figure 2:**
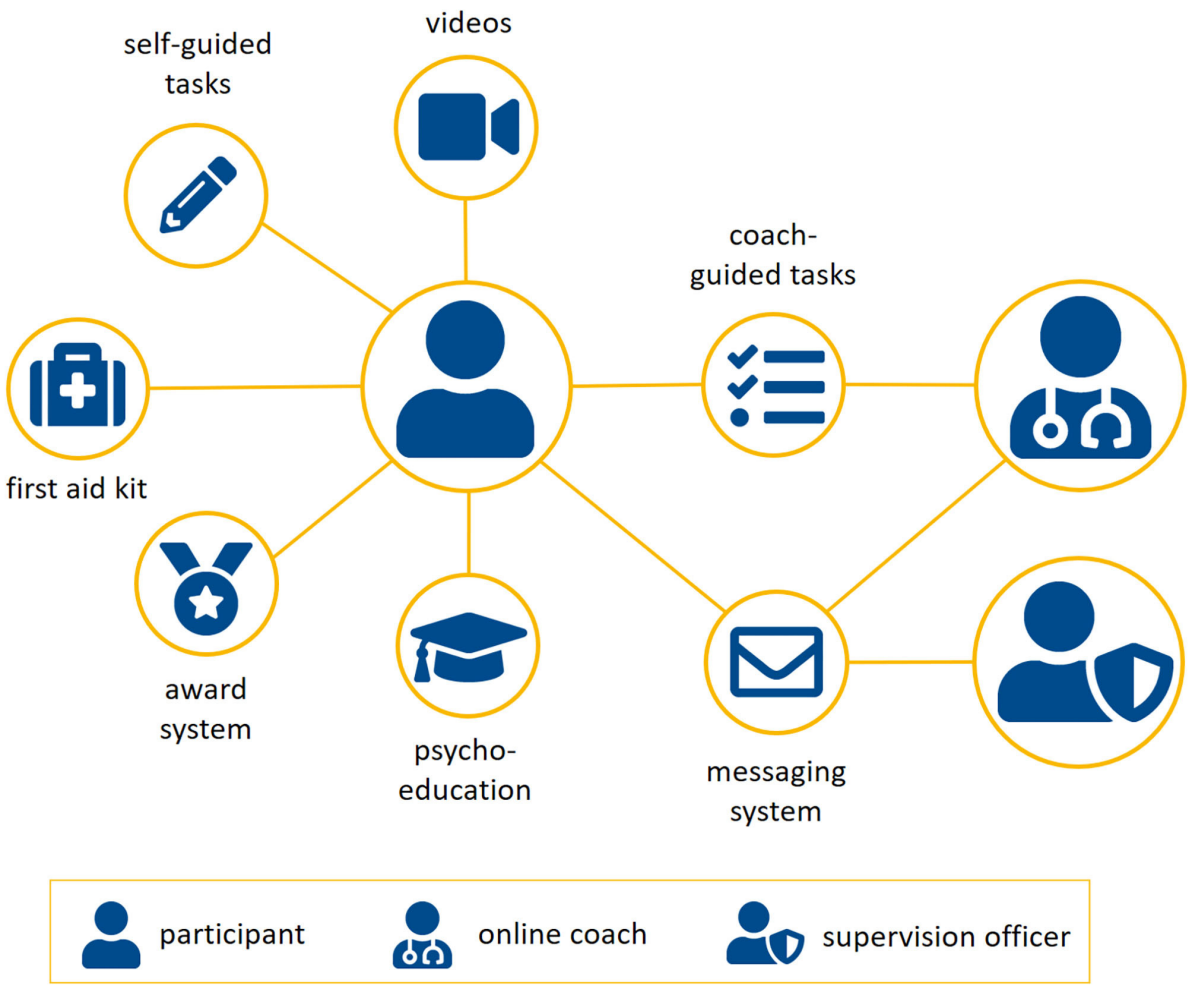
Overview of the contents of the experimental condition. Besides of multimedia psycho-educative elements, exercises are provided in order to train and internalize most important aspects of the psycho-educative elements. Some exercises are coach-guided. An in-app messaging system allows participants to communicate with their online coach or supervision officer in real-time. An automatic reward system is implemented based on a gamification approach. A first aid kit is available for each participants filled with skills for critical situations.

Psychological treatment in the context of individuals who have sexually offended is most effective when it follows the RNR approach utilizing cognitive-behavioral treatment techniques (9). The RNR approach highlights the fact that effective treatment programs have to concentrate on risk factors, which are changeable (7). Thus, most important for effective treatment frameworks are risk factors with empirical evidence for recidivism and for their changeability by psychological treatments (15). Mann et al. identified empirically supported risk factors for ISAC based on meta-analyses, which build the theoretical basis for the web-based intervention modules. Reviewing treatment techniques, Carter and Mann found that cognitive skills training, cognitive restructuring and experimental techniques are empirically supported (6). Following this rationale, the web-based intervention concentrates only on empirically supported risk factors as well as on treatment methods that have empirical support for the respective risk factors. All web-based intervention modules use already developed therapeutic concepts and are partly based on already established community-based treatment programs (48, 49). These concepts have been adapted for web-based interventions and the specific sample of the trial. Beside of treatment techniques, therapist-client relationship is important: a confrontational therapy style, as proposed in some treatment programs, is obstructive and less effective than an emphatic therapist-client relationship following the principles of motivational interviewing (6). Thus, all web-based intervention modules comprise techniques of motivational interviewing (50) to enhance the motivation of the participants, psycho-educational modules, online training, and tasks in order to learn and practice new ways of thinking and coping. Throughout all modules, the speech is friendly, empathetic, simple, and easy to understand (e.g., no technical terms, examples of everyday life) in order to account for the different cognitive functioning level within the group of convicted ISAC and ICCSEM. Following this rationale, the web-based intervention is conceptualized as a structured intervention which comprises six modules in a fixed order, each targeting different risk factors (see Figure 1 for an overview). The content is provided online by psycho-educational blocks complemented by short videos and images. Eighteen different online exercises (e.g., fill-in-the-gap, multiple choice questions) are used to enable the user to train and to internalize the most important aspects of the psycho-educational blocks. The exercises are provided as self-guided tasks with automatic feedback and as coach-guided tasks, where the feedback is provided by online coaches. The feedback of the online coaches is manualized. Since guided web-based interventions are more effective than pure self-help programs, coach-guided exercises are one possibility to enhance the effectiveness of web-based interventions (37). Virtual participants accompany the participant, talk/narrate about their experiences, and make suggestions from the perspective of someone who is also affected. Furthermore, an in-app messaging system allows the user to communicate in real-time with the online coach as well as the SO. In order to enhance the extrinsic motivation of participants, an automatic reward system is integrated based on a gamification approach. That is, participants receive virtual awards as well as coins for each finished block and filled out questionnaire. The coins can be changed into Euros and when the participant has collected at least 20 Euros, he can request the payout online. The participant can receive a maximum compensation of 120 euros. Finally, an online first aid kit is provided for each user and filled with individual skills prepared by the user itself during the web-based intervention. See Figure 2 for an overview of the intervention concept.

#### 2.3.1. Motivation Module

Ward et al. name offender's motivation to change and the resulting involvement in therapy as an intermediate target to be addressed before criminogenic needs can be changed (51). A study surveying ISAC showed a positive impact of motivation for treatment on therapy success (52). In a web-based program for the treatment of alcoholism, motivation to change correlated with dropout out of the program as well (53). Furthermore, Melville et al. illustrated in a review article that most patients who abort a web-based intervention do so before the actual treatment begins (54). Van Ballegoojen et al. come to a similar conclusion, adding that regarding the point in time at which treatment is abandoned, there is little difference between outpatient therapies and web-based therapist-guided treatment (55). As it is reasonable to expect that for convicted ISAC and ICCSEM under CS, motivation to change has an effect on treatment progress, -success and dropout too (52, 56), this underlines the necessity of working on participant's motivation at the beginning of the planned study. The motivation module makes use of motivational interviewing techniques (50). They are based on the assumption that people are ambivalent about changing their behavior. The therapist should support the patient in finding both reasons for and against changing their behavior, thus allowing the patient to naturally develop a desire to change. Accordingly, working with values and goals is a central aspect of motivational interviewing. The four principles underlying this process are (1) express empathy, (2) develop discrepancy, (3) avoid arguments, and (4) support self-efficacy. The methods used to implement these principles are (1) asking open questions, (2) reflective listening, (3) appreciating statements, (4) methods supporting change talk, (5) methods dealing with resistance, (6) methods supporting confidence talk, and (7) summaries.

#### 2.3.2. Supervision and Social Relationships Module

Theories about criminal conduct emphasize the high relevance of social influences for recidivism (57). Negative social influences have been shown to be a valid predictor for sexual re-offenses and are mentioned as a psychological meaningful risk factor (15). Another risk factor for sexual re-offenses can be described as resistance to rules and supervision (15, 58). Thus, one can hypothesize that effective treatments for convicted ISAC and ICCSEM under CS should support the setup of a social network, which reduces the risk to re-offend. Such a positive social network comprises not only the family, friends, or colleagues but also therapists, SOs and social workers. In the supervision and social relationships module, @myTabu tries to enhance the motivation to build up a (new) positive social network and to reduce resistance to rules and supervision with the help of psycho-educative and cognitive-behavioral techniques; e.g., the correlation between re-offenses and a negative social network are demonstrated and techniques to setup new positive networks and to disassociate from negative social influences are provided. Another important risk factor to re-offend is described as a lack of emotionally intimate relationships with adults (15). Individuals who have sexually offended who are able to maintain a stable partnership with adults showed lower recidivism rates in comparison to individuals who have sexually offended who are not able to maintain stable partnerships to adults (58, 59). Furthermore, Hanson et al. demonstrated a linear correlation between a lack of emotionally intimate relationships with adults and re-offenses (60). In consequence, the risk to re-offend of convicted ISAC and ICCSEM under CS is lower if they maintain emotionally intimate relationships with adults. During the supervision and social relationships module, knowledge about this correlation and support for setting up emotionally intimate relationships with adults is provided. To the best of our knowledge, there exist no studies which have demonstrated the effectiveness of the described content of the supervision module, whether in f2f nor in web-based interventions.

#### 2.3.3. Emotion Management Module

Mann et al. defined lifestyle impulsiveness as low self-control, instability in employment and housing, lack of meaningful daily routines, irresponsible decisions, as well as limited and unrealistic long-term goals (15). Lifestyle impulsiveness is mentioned as an empirically supported risk factor with a broad empirical basis (15, 58). Dysfunctional coping, defined as sexualized coping and external coping styles, is not as well-empirically supported as lifestyle impulsiveness (15). Nevertheless, Knight and Thornton showed that external coping, defined as the tendency to react in a reckless and impulsive manner to stressful and emotional negative events, predicts sexual recidivism (61). @myTabu utilizes Dialectic Behavioral Therapy [DBT; (62)] skills training and Acceptance and Commitment Therapy [ACT; (63)] in order to reduce impulsiveness and to enhance coping abilities during stressful or negative emotional life-events. DBT skills training aims to enhance emotion regulation and distress tolerance by teaching several skills which can be used instead of external or sexualized coping. It has already been shown that DBT is successful in reducing impulsive behavior in forensic populations (64). With regard to other psychiatric disorders, web-based interventions following the principles of DBT have been shown in meta-analyses as effective, especially in reducing stress (65, 66). ACT focuses on the development of a meaningful life by accepting negative inner experiences as they appear and provides additional useful strategies to cope with negative emotions (63). Also ACT has been successfully implemented into offender therapy [e.g., (67)] and shows encouraging results if utilized in web-based interventions for a number of psychiatric disorders (65, 66).

#### 2.3.4. Problem Solving Module

In meta-analyses, poor (social) problem solving is significantly linked to sexual recidivism (15, 68). Social problem solving can be defined “as the self-directed cognitive-behavioral process by which an individual, couple, or group attempts to identify or discover effective solutions for specific problem encountered in everyday living” (69). Within the widely-accepted cognitive model of D'Zurilla and Goldfried, problem solving is seen as two-dimensional: the problem orientation and the problem-solving style. Regarding problem orientation, positive (PPO) and negative problem orientations (NPO) can be observed; PPO facilitates adaptive coping and reduces emotional distress, whereas NPO enhances avoidance behavior and ineffective coping strategies (70). Following the theoretical model, three problem-solving styles can be identified: Rational Problem-Solving (RPS), Impulsive Coping Style (ICS), and Avoidance Style (AS). RPS refers to a systematic and planned application of skills leading to an adaptive solution. ICS is unsystematic and mostly results in implementing the first idea that comes in mind, and AS results in avoiding problems and waiting for external solutions. It has been shown that ISAC displayed higher levels of NPO and lower levels of PPO than the general population. In addition, ISAC tend more to ICS and AS than to RPS (71). Furthermore, there is empirical evidence that poor problem solving of ISAC can be effectively changed by psychological treatment techniques (72, 73). The problem solving module is intended to improve poor problem solving skills by following the well-established Problem-Solving Therapy [PST; (74)]. The focus is on the establishment and training of positive problem orientation and rational problem solving skills.

#### 2.3.5. Offense-Supportive Attitudes Module

Offense-supportive attitudes of ISAC can be summarized as the belief that children are able to build sexually mature relationships, that children are not harmed by sex with adults, and that children consciously provoke sexual behaviors in adults (75–77). These cognitive schemata have to be strictly confined from excuses and attempts of justifying the own specific offenses (15). Helmus et al. demonstrated in a meta-analysis that only offense-supportive attitudes as defined above are a psychological meaningful risk factor in the sense of being predictive for sexual recidivism in ISAC. On the other side, attempts of justifying the own offenses should not be the focus of cognitive treatment of ISAC (78). The offense-supportive attitudes module utilizes cognitive restructuring in order to change offense-supportive attitudes. Cognitive restructuring is the most common and best evaluated technique to change the cognition of ISAC (75). Changing implicit theories, attitudes and beliefs is traditionally difficult, especially in the case of ISAC, since the implicit attitudes are not always harmful for the participant and, in contrast, can serve as a strategy to minimize the harm elicited by own offenses (75). Nevertheless, it has been shown in several studies that cognitive restructuring is effective in changing implicit offense-supportive beliefs and attitudes of ISAC (79, 80). Cognitive restructuring assumes that implicit cognitive theories can be changed by identification and logical re-examination. First, the concept of implicit offense-supportive beliefs is explained and individual distorted beliefs are identified by e.g., re-thinking the offense chain. Second, the identified beliefs are re-framed as offense-supportive and as a hindrance for reaching primary goals. Third, the participant is assisted and motivated to evaluate the identified beliefs with regard to rationality and evidence. By using the socratic dialog technique, the participant is not educated but rather supported to find more adequate, alternative ideas about his beliefs. Fourth, these alternative ideas are excessively compared with the old problematic beliefs in several life situations regarding their evidence (75, 79). To the best of our knowledge, @myTabu utilizes cognitive restructuring for ISAC in a web-based intervention for the first time. Thus, no existing data can be provided with regard to effectiveness of online cognitive restructuring of ISAC. But, several meta-analyses for other psychiatric disorders, e.g., depression, have demonstrated that cognitive restructuring techniques integrated in web-based interventions show medium to large effect sizes (81). It seems to be most important for the effectiveness of this web-based intervention module that it is online-guided by therapists (82).

#### 2.3.6. Sexuality Module

Individuals who have sexually offended have been shown to be more sexually active and interested in sexuality than non-offenders (83). Sexual preoccupation is expressed by sexual thoughts and behavior, which are difficult to control or can not be controlled at all (84). Studies demonstrated that sexual preoccupation is highly correlated with sexual re-offenses (58). Thus, one goal of the sexuality module is to provide basic knowledge about sexuality and to support the reduction of excessive sexual thoughts and activities. Furthermore, an awareness for risky situations that may foster sexual thoughts and behavior is created. Techniques to avoid sexual thoughts and activities are provided in order to reduce sexual preoccupation. Emotional congruence with children is another risk factor targeted in the sexuality module. Convicted ISAC and ICCSEM under CS sometimes have the feeling of being emotionally connected to children. Convicted ISAC and ICCSEM under CS, who identify themselves with children, are getting easier in contact with children (84). Thus, emotional congruence with children is one factor highly associated with re-offenses (58). The sexuality module aims to reduce the emotional congruence with children by psycho-educative techniques. Participants should learn that equal relationships between children and adults are not possible. Furthermore, strategies are provided in order to establish a higher (emotional) distance to children. Sexual interests in (and sexual fantasies about) children is one of the most important risk factors for convicted ISAC and ICCSEM under CS (15, 16, 58, 59) and seems to be a motive for sexual child abuse (85). In order to reduce the risk to re-offend, it may be promising to decrease the sexual interest in children and to spotlight non-deviant sexual interests. It is an ongoing debate in literature, whether sexual interests can change throughout the life-time and there exist not enough empirical evidence to answer this question clearly yet (16). From a clinical point of view, changes of sexual interests are reported as well as highly fixated pedophilic interests, which are not changing over the life-time (16). Sexual interests in children is described as a dynamic risk factor that may or may not change over time. Sexual fantasies about children are illustrated as a risk factor for re-offenses. Cognitive-behavioral techniques are used to influence sexual interest in and, if possible, sexual fantasies about children and to enhance sexual interest in adults. The above described techniques used in the sexuality module have been shown to be effective in f2f treatments (86, 87). Also, at least for young people, some techniques have already been used in web-based interventions (88, 89).

### 2.4. The Placebo Web-Based Intervention (Placebo Condition)

The control condition is a placebo web-based intervention with the same mode, dose, and amount of support and attention by supporting online coaches as in the experimental condition—but the content of the modules is unrelated to the proposed dynamic risk factors. The content of the placebo condition comprises information on healthy living, e.g., healthy nutrition, physical activities, or healthy sleep. The amount of exercises is the same as in the experimental condition but exercises are specifically designed to not induce any sustainable effect.

### 2.5. Outcome Measures

In a 5-years follow-up, officially recorded re-offenses are assessed and analyzed for both treatment arms. In order to control for inter-individual pre-treatment scores and risk-levels, the pre-treatment scores are included as co-variables in the model for the primary endpoints. Following the recommendations of Olver and Wong as well as Olver and Stockdale, treatment change is also measured by risk assessment tools and questionnaires (16, 17).

#### 2.5.1. Primary Outcome Measures

Primary outcome measures comprise officially recorded re-offenses and self-report questionnaires assessing dynamic risk factors.

##### 2.5.1.1. Officially recorded re-offenses

Officially recorded re-offenses in the experimental condition compared to the placebo condition are the first primary outcome. Officially recorded re-offenses are obtained 5 years after last patient out.

##### 2.5.1.2. Index of Desistance (IoD)

The second primary outcome measure is the pre-post intervention difference of the Index of Desistance (IoD) in the experimental condition compared to the placebo condition. The IoD represents the individual risk to re-offend assessed through self-report. This composite measurement reflects improvements with regard to psychological meaningful risk factors, offense behaviors as well as offense-related behaviors that have not been registered or convicted yet. The IoD comprises three self-report instruments, which were developed and are currently evaluated by members of the @myTabu-consortium: (1) The Acute-2007-SR is an online and self-report adaption of the Acute-2007 (90) risk-assessment tool in order to assess treatment induced short-term changes in recidivism risk factors. (2) The Checklist of Treatment Effectiveness (CTE) is developed to assess the differential treatment-induced changes in every individual treatment module. Thus, the CTE assesses the constructs that are targeted by the six modules of the invention. (3) The Checklist of Behavioral Misconduct (CMC) measures offense-related behavior as well as low- and high-level new offenses. Offense-related behavior includes behaviors which are relevant for individuals previously convicted of sexual offenses prior to a new offense (e.g., recidivistic behavior like, e.g., if person convicted of sexual abuse of minors visits a playground or a school). The CMC could be seen as post-treatment deviant behavioral indicators. Please see Supplementary Table 1 for the specific measurement time-points. The IoD will provide the individual risk level to re-offend by summing up the scores of the Acute-2007-SR, CTE, and CMC.

#### 2.5.2. Secondary Outcome Measures

Pre-post difference scores of psychometric questionnaires in the experimental condition compared to the placebo condition are defined as the secondary outcomes. The questionnaires are assessed before and after each corresponding intervention module. This approach allows evaluating each web-based intervention module individually with regard to its targeted risk factors. Supplementary Table 1 gives an overview over all questionnaires and the respective assessment time-points.

##### 2.5.2.1. Corrections Victoria Treatment Readiness Questionnaire (CVTRQ)

The Corrections Victoria Treatment Readiness Questionnaire [CVTRQ; (91)] is a self-report measure designed to assess treatment readiness in offenders who have been referred to a cognitive skills program. It comprises 20 items and consists of four scales: (1) Attitudes and motivation, (2) emotional reaction, (3) offending beliefs, and (4) efficacy. Each item has to be answered on a five-point Likert-scale. The CVTRQ shows an acceptable convergent validity, discriminant validity as well as predictive validity (91). To the best of our knowledge, there is no validated German version of the CVTRQ. @myTabu translated the CVTRQ. The translation was checked and translated back into English. Resulting differences due to adaptation were inspected and discussed in terms of item content.

##### 2.5.2.2. Readiness to Change Questionnaire (RCQ)

The Readiness to Change Questionnaire [RCQ; (92)] is a 12-item questionnaire originally designed to identify the stage of change reached by individuals who excessively drink alcohol. The German version [RCQ-D; (93)] was adapted within the project by changing the questions regarding drinking of alcohol in questions regarding the offenses of ISAC and ICCSEM. Responses are made on a five-point Likert-scale. The RCQ-D as well as the RCQ shows good psychometric properties (93). Psychometric properties of the version adapted for ISAC and ICCSEM are not available yet.

##### 2.5.2.3. Optimized Questionnaire for the Measurement of Psychological Reactance (OQMPR)

The Questionnaire for the Measurement of Psychological Reactance [QMPR; (94)] is a questionnaire for the assessment of psychological reactance defined as the theory that people resist attempts to constrain either their thoughts or their behaviors. The Optimized Questionnaire for the Measurement of Psychological Reactance [OQMPR; (95)] is a German variant of the QMPR. The OQMPR consists of 12 statements on a five-point Likert-scale. It has a test-retest-reliability of *rtt* = 0.85 (95).

##### 2.5.2.4. Social Support Questionnaire (F-SozU K-7)

The seven-item short version of the Social Support Questionnaire (F-SozU K-7) is an efficient questionnaire to assess perceived social support (96). Each item comprises a five-point Likert-scale. Internal consistency of the original long version showed Cronbach's α between 0.81 and 0.93 (97).

##### 2.5.2.5. University of California Los Angeles Loneliness Scale (UCLA)

The UCLA Loneliness Scale (98) consists of 20 items in order to assess subjective feelings of loneliness. The German short version (99) consists of 12 items on a four-point Likert-scale. A German study with ISAC and ICCSEM demonstrated a high reliability of α = 0.92 (100).

##### 2.5.2.6. Bumby Molest Scale (BMS)

The Bumby Molest Scale [BMS; (76)] is a self-report questionnaire which assesses cognitive distortion in ISAC [German Version: (101)]. It consists of 38 items on a four-point Likert-scale. The German version shows good construct validity, internal consistency, and test-retest reliability (101).

##### 2.5.2.7. Social Problem-Solving Inventory Revised (SPSI-R)

The Social Problem-Solving Inventory Revised [SPSI-R; (102)] is a self-report questionnaire for the assessment of the five dimensions in the social problem-solving model: (1) positive problem orientation, (2) negative problem orientation, (3) rational problem solving, (4) impulsivity/carelessness style, and (5) avoidance style. The SPSI-R consists of 52 items on a five-point Likert-scale. It shows good psychometric properties for individuals who have sexually offended (72). The validated German version is a short form consisting of 25 items (103).

##### 2.5.2.8. Difficulties in Emotion Regulation Scale—Sub-scale Impulsivity (DERS)

The Difficulties in Emotion Regulation Scale [DERS; (104)] is a self-report questionnaire to assess emotion dysregulation. The sub-scale “impulse control difficulties” is used as outcome measure and comprises five items on a five-point Likert-scale. The German version is used, which shows a good internal consistency, construct and predictive validity (105).

##### 2.5.2.9. Negative Affect Repair Questionnaire (NARQ)

The Negative Affect Repair Questionnaire [NARQ; (106)] is a self-report questionnaire to assess strategies to regulate negative affect in a systematic manner. It consists of 17 items on a five-point Likert-scale and provides a good construct validity. Reliability scores (Cronbach's α) for the three NARQ scales ranged between 0.71 and 0.80 (106).

##### 2.5.2.10. Barratt Impulsiveness Scale-15 (BIS-15)

The Barratt Impulsiveness Scale [BIS; (107)] is a questionnaire developed to assess the personality/behavioral construct of impulsiveness. It consists of 30 items on a four-point Likert-scale. The German short version [BIS-15; (108)] consisting of 15 items is used in the clinical trial. The BIS-15 is an efficient measure of impulsiveness with good internal consistency [α = 0.81; (108)].

##### 2.5.2.11. Coping Using Sex Inventory (CUSI)

The Coping Using Sex Inventory [CUSI; (109)] is a questionnaire to assess the presence of and the degree to which sex was used to deal with problematic situations. It consists of 16 items on a five-point Likert-scale with a satisfying internal consistency (109). The translation was checked and translated back into English by the authors. Resulting differences due to adaptation were inspected and discussed in terms of item content. For another German translation, it has been shown that the CUSI is able to assess therapy-induced changes of ISAC (2).

##### 2.5.2.12. Hypersexual Behavior Inventory-19 (HBI-19)

Sexual preoccupation is assessed by the Hypersexual Behavior Inventory-19 [HBI-19; (110)]. The HBI-19 is a three-factor measure (coping, control, and consequences) developed to assess hypersexual behavior. The instrument consists of 19 items (e.g., “I use sex to forget sorrows of everyday life.”) answered on a scale from 1 (never) to 5 (very often). The maximum score is 95, with higher scores indicating a higher level of sexual preoccupation. The questionnaire was shown to have good reliability (α = 0.90) and validity (110).

##### 2.5.2.13. Questionnaire on Emotional Congruence with Children-Revised (EKK-R)

Emotional congruence with children is assessed by the Questionnaire on Emotional Congruence with Children-Revised [EKK-R; (111)] including three factors (special relationship to children, immaturity, and emotional closeness to children). Twenty items are answered on a four-point scale. The questionnaire demonstrated good reliability (α = 0.80) and validity (111).

##### 2.5.2.14. Specific self-efficacy for modifying Sexual Interest in Children (SSIC)

Specific self-efficacy for modifying sexual interest in children (SSIC) is assessed by the Self-Efficacy for Modifying Sexual Interest in Children Scale [SSIC-Scale; (112)]. Six items on the participant's conviction regarding the ability to change their sexual interest in children (e.g., “I can succeed in reducing my sexual interest in children”) were answered on a scale from 1 (do not agree at all) to 5 (totally agree). The maximum score is 30, with higher scores indicating a higher level of self-efficacy. The instrument was shown to have good reliability (α = 0.87) and validity (112). Four variables that were shown to be related to the SSIC (113, 114) are also assessed: flexibility of sexual interest in children (three items on previous experiences concerning changes in the participant's sexual interest in children), exclusiveness of sexual interest in children (single-item question), motivation to change sexual interest in children (single-item question), and age of onset of sexual interest in children (single-item question).

##### 2.5.2.15. The Explicit Sexual Interest Questionnaire (ESIQ)

The Explicit Sexual Interest Questionnaire [ESIQ; (115)] directly assesses pedophilic interest. It consists of two scales measuring sexual behavior (20 items, e.g., “I enjoyed orally stimulating a man.”) and sexual fantasies (20 items, e.g., “I find it attractive to imagine a little boy sexually stimulating me.”). All items are answered on a scale from 1 (totally disagree) to 5 (totally agree). The reliability of the instrument ranges between 0.86 and 0.97 (115).

##### 2.5.2.16. Outlet Inventory-Revised (SOI-R)

The second measurement for sexual interest in children is the Item 2a of the Sexual Outlet Inventory-Revised [SOI-R; (116)] which assesses the desire for sexual activity involving children on a visual analog scale from 0 (desire is absent) to 100 (I have to act to satisfy the desire). Higher values on the scale indicate a stronger sexual interest in children (116).

#### 2.5.3. Additional Questionnaires

Some questionnaires will be administered additionally but have no direct explanatory power with regard to primary or secondary outcomes. There are no concrete hypotheses regarding secondary or primary outcome. Questionnaires are administered for descriptive purposes, security reasons or to control for possible bias effects (e.g., volunteer-effects).

##### 2.5.3.1. Working Alliance Inventory-Short Revised (WAI-SR)

The WAI-SR (117, 118) measures three dimensions of therapeutic alliance: (a) bond, (b) task, and (c) goal. For the purpose of this study, the original items of the German version (119) were adapted to be suitable for a web-based intervention. The items have to be rated on a five-point Likert-scale ranging from 1 (never) to 5 (always). It was adapted to suit to the online-coach as well as the SO resulting in 16 items. Internal consistency (Cronbach's α) of the original questionnaire ranges from 0.82 to 0.90 (119).

##### 2.5.3.2. Questionnaire of Subjective Therapy Preconditions (SBV-R)

The SBV-R (120) measures the multidimensional construct of therapy and treatment preconditions in offenders. In this study, the only sub-scale used is “pressure of pain.” The pressure of pain is defined as an aversive state in which the person affected recognizes conflicts as lying within him or her (121). The eight items of this sub-scale are rated on a five-point scale. The internal consistency (Cronbach's α) of the sub-scale pressure of pain is 0.75 (120).

##### 2.5.3.3. WHO-5 Well-Being Index (WHO-5)

The WHO-5 (122) is a questionnaire that measures current mental well-being with five items. The items are rated on a six-point Likert-scale ranging from 0 (at no time) to 5 (all of the time). The WHO-5 has shown good validity in measuring subjective well-being in clinical studies (123).

##### 2.5.3.4. Questionnaire for Acceptance of Technology (ATQ)

To assess acceptance of technology and its predictors within the framework of this project, a questionnaire for acceptance of technology (ATQ) was designed. The ATQ is based on the Unified Theory of Acceptance and Use of Technology (UTAUT) which defines acceptance as behavioral intention of using a certain technology. According to the model, predictors of this intention are performance expectancy, effort expectancy, and social influence. Predictors of actual technology usage are facilitating conditions, such as organizational or technical preconditions (124). The ATQ comprises items which measure acceptance and the previously named predictors from the UTAUT model as well as additional items concerning following extended predictors: internet anxiety, attitude toward using technology, planning, and importance of monetary compensation. The items of the ATQ were either self-constructed or derived from questionnaires used in former studies and adapted if conceptually required (125–127). Internal consistency (Cronbach's α) of the original scale ranges from 0.68 to 0.92. To control for effects of compensation, two items concerning importance of study compensation were self-constructed.

##### 2.5.3.5. Mood and Risk Questionnaire (MRQ)

The Mood and Risk Questionnaire (MRQ) was developed to assess acute changes with regard to emotional well-being and thoughts associated with potential re-offenses as a direct cause of working on sessions within the web-based intervention. It consists of ten self-developed items, of which six are asking about thoughts and behaviors associated with potential re-offenses and four asking about the psychological and emotional state of the participant. The MRQ is provided before and after each session.

##### 2.5.3.6. Questionnaire of Non-Participation (QNP)

The Questionnaire of Non-Participation (QNP) was developed to assess reasons for not taking part in the study. It comprises eleven items on a six-point Likert-scale. Items are e.g., “I will not take part in the study because I do not need any further help.” or “I will not take part in the study because I am afraid of negative consequences if my SO gets additional information about me.” The QNP will only be administered if a potential participant will not take part in the study but has given informed consent to fill out this questionnaire.

### 2.6. Procedure

The clinical trial starts on January 01, 2021 and ends on December 31, 2022. All potential participants are screened by their SO based on inclusion criteria and the SO hands out written information on the trial. If the potential participant is generally interested in participating, an individual meeting with an independent research assistant will take place at the supervision office. During this meeting, the research assistant provides detailed information about the goals and procedure of the trial, data security and advantages or disadvantages of taking part in the trial. If the potential participant is further interested to take part, he or she will receive an information letter, the informed consent, an intervention agreement, and a letter releasing the SO from the confidentiality obligation. In the intervention agreement, the participant agrees not to commit offenses during the participation and is informed that any violation will lead to exclusion. Furthermore, the research assistant performs the pre-intervention assessments (see Supplementary Table 1). If a potential participant does not want to take part in the trial, he or she will be asked for informed consent in filling out one questionnaire (QNP) and to allow to fill out the SSQ-CF checklist by the research assistant as well as the SSQ-SO by the SO. Further, the potential participant is asked for allowance to assess his or her officially registered re-offenses in a 5-years follow up in order to be able to control for a potential volunteer-effect. After obtaining the written informed consent, the intervention agreement, and the release from confidentiality, the participant is randomly allocated to the experimental or the control condition. The username as well as the initial password is delivered by post to the SO, who will give it to the participant. After the first log-in, the participant has to change the initial password due to security reasons. After changing the initial password, the participant has to go through an introduction, which explains the usage of the intervention website, before he or she can start with the first online module. The procedure for the placebo condition is the same as for the experimental condition, only the provided online contents differ. In both the placebo and the intervention condition, the provided content is divided into six online modules. Each module is further divided into four sessions. Each session has a duration of about 60 min and the participant is advised to finish one session per week. Consequently, the intervention as well as placebo consists of 24 sessions which can be completed in 24 weeks. The participant will be reminded by in-app messages or by the SO if he or she is late in finishing the next session. During both online conditions, data of primary outcome (IoD) is assessed at 7 measurement time-points. Except questionnaires which are filled out at clearing up all questionnaires are provided online within the web-based app. This ensures that the questionnaires are filled in at the predefined measurement time-points. Five years after the last patient out, re-offenses are assessed by officially documented re-offenses (see Supplementary Table 1 for an overview on measurement time-points).

### 2.7. Randomization

The trial comprises two parallel groups (web-based intervention group and web-based placebo group). Subjects are randomly allocated to treatment using an allocation ratio of 1:1. The randomization lists are centrally generated using a computerized system (SecuTrial® www.secutrial.com, a for-profit organization) stratified by the offense type (§§176, 176a, 176b StGB vs. §184b StGB), type of CS (§§56, 57 StGB vs. §§68 ff StGB), treatment in addition to CSaU (external psycho-therapeutic and/or medical treatment vs. no treatment), and risk to re-offend at baseline [assessed with the modified Static-99 (43) for ICCSEM and the Static-99 (42) for ISAC]. At screening, each subject receives the next consecutive screening number. At randomization, each subject eligible for study participation receives the next consecutive randomization number according to his stratum from a block of randomization numbers (block size: 4).

### 2.8. Blinding

Due to security reasons, blinding of SOs and supporting coaches is not possible. Nevertheless, participants, members of the data safety monitoring board (DSMB), and data analysts (observer) are blinded until the end of the data analysis. The DSMB is independent from the sponsor.

### 2.9. Adherence of Therapists to the Protocol

Feedback to participants is automatized and otherwise manualized. Regular meetings between online coaches and an experienced supervisor will promote treatment consistency and regular supervision (one meeting per month).

### 2.10. Power and Sample Size Calculations

It is assumed that participants who take part in the experimental treatment show significantly lower values of self-reported dynamic risk factors inter-, and post-treatment controlling for baseline values as well as less officially recorded re-offenses in a 5-years follow-up, compared to the placebo condition. Within programs for individuals who have sexually offended, higher levels of motivation and better treatment engagement were associated with decreased attrition (128). The study design addresses this in a direct manner by providing an online motivation module at the beginning of the intervention. A rate of 50% of screened participants taking voluntarily part in the clinical trial is assumed. Based on the estimation of the ministry of Lower-Saxony and Baden-Wuerttemberg, it is expected to see 1,165 subjects eligible for participation during the recruitment period of which 50% (*N* = 582) are expected to participate in the study. Taking a dropout of 26.4% (128) into account, it is expected to see *N* = 428 subjects completing the web-based intervention or placebo intervention. With *N* = 214 subjects in both conditions (treatment and placebo) it is possible to detect small effects (*d* = 0.27) with a power of 80% at a significance level α = 5%. This effect size is in the expected range based on the above shown literature.

### 2.11. Statistical Analysis

All patients who discontinue from the study are identified and the extent of their participation in the study is reported. If discontinuation is due to clear evidence for recidivism or concrete preparation for or intention to recidivism (assessed by responsible SO or guiding therapist) the subject receives the maximal value for self-reported dynamic risk factors for the remaining measurements (assuming the worst case of a failed treatment). In all other cases of drop-out, the reason is recorded. The efficacy of the web-based intervention based on the first primary outcome measure (IoD) will be analyzed by means of Gaussian linear model for repeated measures (MMRM) with treatment group, time (six time-points; after each web-based intervention module (see Supplementary Table 1); treatment-by-time interaction, and the randomization strata as factors (offense type, type of CS, external treatment in addition to CSaU), and baseline measurements (at clearing up) of the outcome as co-variate. The error terms are assumed to follow a multivariate normal distribution with unstructured co-variance. Least squares mean changes from baseline are reported for the treatment groups with 95% confidence interval (CI) as well as the difference between the least squares treatment group means with 95% CI and *p*-value testing the null hypothesis of no treatment effect. The analysis is performed on the Intention-To-Treat (ITT) population comprising all patients who have completed at least one module. The second primary outcome measure (officially recorded re-offenses) will be analyzed by means of Cox proportional hazard model with treatment group, number of completed modules and the randomization strata (offense type, type of CS, external treatment in addition to CSaU, risk to re-offend at baseline). The effect of each module will additionally be analyzed using the Wilcoxon signed rank test for paired data comparing the pre- and post-module scores, adjusting the *p*-values using the Bonferroni-Holm's method to control the family-wise error rate. Economic analyses will be based on analytic modeling techniques. Data will be derived from the clinical trial (e.g., recidivism rates based on dynamic risk factors), the literature (e.g., tangible benefits), and experimental procedures (e.g., contingent valuation/revealed preference approaches to assess intangible benefits). Results of the analysis will be presented in terms of net benefit (NB) estimates and a benefit-cost ratios. The NB will be calculated as NB = tangible benefits + intangible benefits-intervention costs. The intervention will be considered cost-beneficial relative to the control condition if the NB is positive and the benefit-cost ratio exceeds 1.00 following standard economic decision rules. Since many cost parameters associated with ISAC and ICCSEM occur after the trial period, the cost-benefit of the intervention will be modeled for a 5-years period. Sensitivity analyses will be conducted to examine how the NB will be influenced by variations in model parameters.

### 2.12. Stopping Rules

The individual subject is excluded from the clinical trial if the responsible SO or the online coach reports clear evidence for sexual recidivism or concrete preparations for any sexual recidivism. The clinical trial is stopped if an interim analysis at the mid-point of the project (12 months after first patient in; tested on subjects having completed at least two modules of the web-based intervention) demonstrates: (a) a significantly higher risk to re-offend (based on main outcome and number of reported significant events in the experimental condition compared to the placebo condition); (b) a significantly higher proportion of reported significant events (see a) in the experimental condition than in the placebo condition; (c) a significantly increased proportion of high-risk subjects (defined in terms of the main outcome) compared to the proportion at baseline.

## 3. Discussion

Within the @myTabu consortium, a web-based intervention for convicted ISAC and ICCSEM under CS has been developed. The web-based intervention targets only empirically supported risk factors and may therefore be more efficient than current f2f treatment programs. The intervention is designed as a guided web-based intervention, which has been shown in the context of psychiatric disorders to be more effective than web-based self-help programs [for an overview see (37)]. The main goal of the trial is to evaluate the effectiveness of the web-based intervention in a placebo-controlled randomized add-on study. To the best of our knowledge, the results of the RCT will provide for the first time information about the usability, effectiveness, and economic benefits of web-based interventions in the treatment of convicted ISAC and ICCSEM under CS. It aims further to evaluate the effectiveness of the individual online modules within the intervention program with regard to its ability to reduce psychological meaningful dynamic risk factors. If proven to be effective, the web-based intervention may provide a significant enhancement of the re-integration of convicted ISAC and ICCSEM under CS into society. The intervention can be easily transferred to treatment programs for ISAC or ICCSEM not under CS, e.g., in prisons or high security hospitals. The web-based intervention may provide a low-threshold service for convicted ISAC and ICCSEM under CS, reaching more relevant people (e.g., in rural areas) than current intervention programs. In addition, it likely carries social and economic benefits by reducing victim- and offender-related costs. On account of these advantages, web-based interventions could help to close the current treatment gap.

### 3.1. Limitations

CS of ISAC and ICCSEM in Germany does not include a psycho-therapeutic or medical treatment by default. However, depending on the case and availability of therapists, the court orders an instruction to take part in an external treatment program. Therefore, it is possible that included participants will get additional f2f treatment. This could lead to a potential ceiling effect. Hence, within the clinical trial external treatments are assessed and included in statistical analyses. Since taking part in the study is based on informed consent (and can not be ordered by court until the effectiveness of the web-based intervention has been shown) a volunteer effect can arise. In order to control for a potential volunteer effect, eligible participants who do not want to take part in the study, are asked for informed consent to assess some information consistent with the strata (e.g., prior offenses, modified Static-99 or Static-99) and allowance to assess officially registered re-offenses in the 5-years follow up. Another possible limitation may be the control condition. To control for specific treatment effects, under placebo condition only content is provided which to our knowledge is not able to reduce risk factors. Further, solely online exercises which try to not trigger any internal processes are provided. However, it can not be ruled out that there are (non-specific therapeutic) effects on dynamic risk factors and re-offenses. There is an ongoing discussion which control condition is best for psycho-therapeutic RCTs [for an overview see e.g., (129, 130)]. Due to the fact that mostly psycho-therapeutic placebo conditions can not control for non-specific therapeutic factors and that most psycho-therapeutic treatments contain many components (like the @myTabu web-based intervention) (131), call psycho-therapeutic placebo type controls “pseudoplacebos.” Following this definition, the placebo condition in this study also has to be seen as a pseudoplacebo condition. With regard to self-guided tasks and psycho-educational content provided within both conditions, it is not possible to ensure that participants are not only clicking through the content. This fact is considered by assessing the duration a participant spends on one lesson (each session is subdivided into lessons) which may allow to draw conclusions from the attention allocation to this lesson. However, this measurement controls only for the time spent on a specific lesson not on the engagement during this time. For coach-guided tasks, it is ensured by the coach that the participant is not only clicking through the content.

## Ethics Statement

The studies involving human participants were reviewed and approved by the protocol of this study (Version 1.0.1) has been approved by the medical ethical board of the Human Medical Center Göttingen, Göttingen, Germany (number: 16/2/20). The patients/participants provided their written informed consent to participate in this study.

## Author Contributions

PF, PB, CB, SE, MR, and JM: conceptualization. PF, PB, SE, MR, and JM: funding acquisition. PF, LB, SSch, ST, PB, CB, AL, and JM: methodology. PF: project administration, software, visualization, and writing—original draft. LB, SSch, BS, ST, and CB: resources. PF and BS: supervision. LB, SSch, BS, ST, PB, CB, SE, MR, AL, and JM: review and editing. All authors have read and approved the manuscript.

## Conflict of Interest

The authors declare that the research was conducted in the absence of any commercial or financial relationships that could be construed as a potential conflict of interest. Access to the final trial data-set for investigators is not limited, e.g., by contractual agreements.

## Glossary

**Table:**

AS	Avoidance Style
ATQ	Questionnaire for Acceptance of Technology
BIS	Barratt Impulsiveness Scale
BMS	Bumby Molest Scale
CMC	Checklist of Behavioral Misconduct
CS	Community Supervision
CSaU	Community Supervision as Usual
CTE	Checklist of Treatment Effectiveness
CUSI	Coping Using Sex Inventory
CVTRQ	Corrections Victoria Treatment Readiness Questionnaire
DERS	Difficulties in Emotion Regulation Scale
DSMB	Data Safety Monitoring Board
EKK-R	Questionnaire on Emotional Congruence with Children-Revised
ESIQ	Explicit Sexual Interest Questionnaire
f2f	face-to-face
F-SozU	Social Support Questionnaire
HBI	Hypersexual Behavior Inventory
ICCSEM	Individuals who consumed Child Sexual Exploitation Material
ICS	Impulsive Coping Style
IoD	Index of Desistance
ISAC	Individuals who sexually abused Children
ITT	Intention to Treat
MMRM	Mixed Model Repeated Measures
MRQ	Mood and Risk Questionnaire
NARQ	Negative Affect Repair Questionnaire
NB	Net Benefit
NPO	Negative Problem Orientation
OQMPR	Optimized Questionnaire for the Measurement of Psychological Reactance
PHQ-D	Patient Health Questionnaire
PPO	Positive Problem Orientation
PST	Problem-Solving Therapy
QNP	Questionnaire of Non-Participation
RCQ	Readiness to Change Questionnaire
RCT	Randomized Controlled Trial
RNR	Risk-Need-Responsivity
RPS	Rational Problem-Solving
SBV-R	Questionnaire of Subjective Therapy Preconditions
SO	Supervision Officer
SOI-R	Sexual Outlet Inventory-Revised
SSIC	Specific self-efficacy for modifying Sexual Interest in Children
SSPI-2	Revised Scale for Pedophilic Interest
SSQ-CF	Sample Specification Questionnaire based on Court Files
SSQ-rSO	repeated Sample Specification Questionnaire for the Supervision Officer
SSQ-SO	Sample Specification Questionnaire for the Supervision Officer
SSQ-P	Sample Specification Questionnaire for the Participant
StGB	German criminal code (Strafgesetzbuch)
UCLA	University of California Los Angeles Loneliness Scale
UTAUT	Unified Theory of Acceptance and Use of Technology
WAI-SR	Working Alliance Inventory-Short Revised
WHO-5	WHO-5 Well-Being Index
