# Supplementary material for: @myTabu—A Placebo Controlled Randomized Trial of a Guided Web-Based Intervention for Individuals Who Sexually Abused Children and Individuals Who Consumed Child Sexual Exploitation Material: A Clinical Study Protocol
Source: Front Psychiatry. 2021 Jan 8;11:575464. doi: 10.3389/fpsyt.2020.575464 (PMC7820175; doi:10.3389/fpsyt.2020.575464)
Supplement: Supplementary file 2 [file Data_Sheet_2.pdf]

Supplementary Material

1 SPIRIT SCHEDULE OF ENROLLMENT, INTERVENTIONS, AND ASSESSMENTS.

Table S1. SPIRIT schedule of enrollment, interventions, and assessments. **SSQ-P**: Sample Specifications Questionnaire for the Participant; **SSQ-SO**: Sample Specifications Questionnaire for the Supervision Officer; **SSQ-CF**: Sample Specifications Questionnaire based on the Court File; **PHQ-D**: Patient Health Questionnaire; **SSQ-rSO**: repeated Sample Specifications Questionnaire for the Supervision Officer; **SSPI-2**: Revised Scale for Pedophilic Interests (Seto et al., 2017); **ICD-11-Screener**: ICD-11-Sexuality-Screener (Briken, 2019); **QNP**: Questionnaire of Non-Participation; **Acute-2007-SR**: Self-Report adaption of the Acute-2007 (Hanson and Harris, 2007); **CMC**: Checklist of Behavioral Misconduct; **CTE**: Checklist of Treatment Effectiveness; **MRQ**: Mood and Risk Questionnaire; **official re-offenses**: officially recorded re-offenses assessed five years after last patient out; **CVTRQ**: Corrections Victoria Treatment Readiness Questionnaire (Casey et al., 2007); **RCQ**: Readiness to Change Questionnaire (Hannöver et al., 2002); **F-SozU**: Social Support Questionnaire, short version Dunkel et al. (2005); **EKK-R**: Questionnaire on Emotional Congruence with Children – Revised (Mack and Yundina, 2012); **OQMPR**: Optimized Questionnaire for the Measurement of Psychological Reactance (Herzberg, 2002); **SOI-R**: Sexual Outlet Inventory - Revised (Briken, 2010) **UCLA**: University of California Los Angeles Loneliness Scale (Russell, 1996); **BMS**: Bumby Molest Scale (Feelgood et al., 2009); **SPSI-R**: Social Problem Solving Inventory Revised (D'Zurilla et al., 2002); **DERS**: Difficulties in Emotion Regulation Scale - Sub-scale Impulsivity (Gratz and Roemer, 2004); **NARQ**: Negative Affect Repair Questionnaire (Scherer et al., 2013); **BIS-11**: Barratt Impulsiveness Scale - 11 (Patton et al., 1995); **CUSI**: Coping Using Sex Inventory (Cortoni and Marshall, 2001); **HBI-19**: Hypersexual Behavior Inventory - 19 (Klein et al., 2014); **SSIC-Scale**: Specific self-efficacy for modifying Sexual Interest in Children (Tozdan et al., 2015); **ESIQ**: Explicit Sexual Interest Questionnaire (Banse et al., 2010); **WAI-SR**: Working Alliance Inventory - Short Revised (Horvath and Greenberg, 1989); **SBV-R**: Questionnaire of Subjective Therapy Preconditions (Wilmers et al., 2008); **WHO-5**: WHO-5 Well-Being Index (Bech, 2004); **ATQ**: Questionnaire for Acceptance of Technology. Please note that the follow-up will be performed five years after last patient out.

REFERENCES

Banse, R., Schmidt, A. F., and Clarbour, J. (2010). Indirect measures of sexual interest in child sex offenders: A multimethod approach. *Criminal Justice and Behavior* 37, 319–335

Bech, P. (2004). Measuring the dimension of psychological general well-being by the WHO-5. *Quality of life newsletter* , 15–16

Briken, P. (2010). Sexual Outlet Inventory-Revised

Briken, P. (2019). ICD-Sexualitäts-Screener

Casey, S., Day, A., Howells, K., and Ward, T. (2007). Assessing Suitability for Offender Rehabilitation: Development and Validation of the Treatment Readiness Questionnaire. *Criminal Justice and Behavior* 34, 1427–1440. doi:10.1177/0093854807305827

Cortoni, F. and Marshall, W. L. (2001). Sex as a coping strategy and its relationship to juvenile sexual history and intimacy in sexual offenders. *Sexual Abuse: A Journal of Research and Treatment* 13, 27–43

Dunkel, D., Antretter, E., Fröhlich-Walser, S., and Haring, C. (2005). [Evaluation of the short-form social support questionnaire (SOZU-K-22) in clinical and non-clinical samples]. *Psychotherapie, Psychosomatik, Medizinische Psychologie* 55, 266–277. doi:10.1055/s-2004-834746

D'Zurilla, T. J., Nezu, A. M., and Maydeu-Oliveras, A. (2002). *Manual for the Social Problem-Solving Inventory-Revised* (North Tonawanda: Multi-Health Systems)

Feelgood, S., Schaefer, G., and Hoyer, J. (2009). KV-M-Skala zur erfassung kognitiver verzerrungen bei missbrauchern

Gratz, K. L. and Roemer, L. (2004). Multidimensional assessment of emotion regulation and dysregulation: Development, factor structure, and initial validation of the difficulties in emotion regulation scale. *Journal of psychopathology and behavioral assessment* 26, 41–54

Hannöver, W., Thyrian, J. R., Hapke, U., Rumpf, H.-J., Meyer, C., and John, U. (2002). THE READINESS TO CHANGE QUESTIONNAIRE IN SUBJECTS WITH HAZARDOUS ALCOHOL CONSUMPTION, ALCOHOL MISUSE AND DEPENDENCE IN A GENERAL POPULATION SURVEY. *Alcohol and Alcoholism* 37, 362–369. doi:10.1093/alcalc/37.4.362

Hanson, R. K. and Harris, A. (2007). *Acute-2007 Scoring Guide* (Ottawa: Public Safety Canada)

Herzberg, P. Y. (2002). Zur psychometrischen Optimierung einer Reaktanzskala mittels klassischer und IRT-basierter Analysemethoden. *Diagnostica*

Horvath, A. O. and Greenberg, L. S. (1989). Development and validation of the working alliance inventory. *Journal of counseling psychology* 36, 223

Klein, V., Rettenberger, M., Boom, K.-D., and Briken, P. (2014). Eine validierungsstudie der deutschen version des hypersexual behavior inventory (HBI). *PPmP-Psychotherapie· Psychosomatik· Medizinische Psychologie* 64, 136–140

Mack, C. and Yundina, E. (2012). Emotionale kongruenz mit der kinderwelt als mögliches diagnostischesMerkmal von pädophilie. *JM Müller, M. Rösler*

Patton, J. H., Stanford, M. S., and Barratt, E. S. (1995). Factor structure of the Barratt impulsiveness scale. *Journal of clinical psychology* 51, 768–774

Russell, D. W. (1996). UCLA loneliness scale (version 3): Reliability, validity, and factor structure. *Journal of personality assessment* 66, 20–40

Scherer, A., Eberle, N., Boecker, M., Vögele, C., Gauggel, S., and Forkmann, T. (2013). The negative affect repair questionnaire: Factor analysis and psychometric evaluation in three samples. *BMC Psychiatry* 13, 16. doi:10.1186/1471-244X-13-16

Seto, M. C., Stephens, S., Lalumière, M. L., and Cantor, J. M. (2017). The Revised Screening Scale for Pedophilic Interests (SSPI–2): Development and Criterion-Related Validation. *Sexual Abuse: A Journal of Research and Treatment* 29, 619–635. doi:10.1177/1079063215612444

Tozdan, S., Jakob, C., Schuhmann, P., Budde, M., and Briken, P. (2015). Spezifische selbstwirksamkeit zur beeinflussung des sexuellen interesses an kindern (SSIK): Konstruktion und validierung eines messinstruments. *PPmP-Psychotherapie· Psychosomatik· Medizinische Psychologie* 65, 345–352

Wilmers, F., Munder, T., Leonhart, R., Herzog, T., Plassmann, R., Barth, J., et al. (2008). Die deutschsprachige version des working alliance inventory-short revised (WAI-SR)-Ein schulenübergreifendes, ökonomisches und empirisch validiertes instrument zur erfassung der therapeutischen allianz. *Klinische Diagnostik und Evaluation* 1, 343–358
